# Coronavirus disease (COVID-19) in a paucisymptomatic patient: epidemiological and clinical challenge in settings with limited community transmission, Italy, February 2020

**DOI:** 10.2807/1560-7917.ES.2020.25.11.2000230

**Published:** 2020-03-19

**Authors:** Emanuele Nicastri, Alessandra D’Abramo, Giovanni Faggioni, Riccardo De Santis, Andrea Mariano, Luciana Lepore, Filippo Molinari, Giancarlo Petralito, Silvia Fillo, Diego Munzi, Angela Corpolongo, Licia Bordi, Fabrizio Carletti, Concetta Castiletti, Francesca Colavita, Eleonora Lalle, Nazario Bevilacqua, Maria Letizia Giancola, Laura Scorzolini, Simone Lanini, Claudia Palazzolo, Angelo De Domenico, Maria Anna Spinelli, Paola Scognamiglio, Paolo Piredda, Raffaele Iacomino, Andrea Mone, Vincenzo Puro, Nicola Petrosillo, Antonio Battistini, Francesco Vairo, Florigio Lista, Giuseppe Ippolito, on behalf of INMI and the Italian Army COVID-19 study groups

**Affiliations:** 1 National Institute for Infectious Diseases ‘Lazzaro Spallanzani’ IRCCS, Rome, Italy; 2 These authors contributed equally to this article and share first authorship; 3 Scientific Department, Italian Army Medical Center, Rome, Italy; 4 Clinical Sciences Department, Italian Army Medical Center, Rome, Italy; 5 Italian Army Surgeon General Office, Rome, Italy; 6 These authors contributed equally to this article and share last authorship; 7 The members of these groups are acknowledged at the end of the article

**Keywords:** COVID-19, paucisymptomatic case, Italy

## Abstract

Data concerning the transmission of the novel severe acute respiratory syndrome coronavirus (SARS-CoV-2) in paucisymptomatic patients are lacking. We report an Italian paucisymptomatic case of coronavirus disease 2019 with multiple biological samples positive for SARS-CoV-2. This case was detected using the World Health Organization protocol on cases and contact investigation. Current discharge criteria and the impact of extra-pulmonary SARS-CoV-2 samples are discussed.

A novel coronavirus, severe acute respiratory syndrome coronavirus-2 (SARS-CoV-2) causing human disease named coronavirus disease (COVID-19) was identified in Wuhan, China, in December 2019 [1]. The number of affected patients rapidly increased and, as at 16 March 2020, more than 165,000 cases were reported (including 6,507 deaths) both in and outside of China [2]. The early identification of imported cases has a crucial role in preventing secondary transmission and it is based on both epidemiological and clinical assessment [3,4]. We report an Italian paucisymptomatic case of COVID-19 with multiple biological samples positive for SARS-CoV-2.

On 3 February 2020, 56 asymptomatic Italian citizens were medically evacuated from Wuhan, China to Rome, Italy for a 14-day quarantine period spent in the Cecchignola military complex. Before leaving Wuhan, all subjects were afebrile and asymptomatic. During the flight, they wore facial masks and proper hand hygiene was prescribed. On 5 February, according to the World Health Organization (WHO) protocol on cases and contact investigation [5], all of them were screened for SARS-CoV-2 RNA by the Scientific Department of the Italian Army Medical Center using real-time RT-PCR on nasopharyngeal swabs. Only one swab resulted positive both for the envelope protein gene E and the RNA-dependent RNA polymerase gene RdRp [6].

## Case description

The individual who tested positive was an Italian man in his late 20s, in healthy condition. He was in Wuhan from 20 January to 3 February to visit his Chinese friends. He had no exposure to marketplaces, live animals, known sick people or access to healthcare facilities. In order to avoid SARS-CoV-2 infection, he stayed at home except for a 1-day walk in the town district. His close contacts were the three members of the Chinese family hosting him, who are still in healthy condition.

On 6 February, the case was admitted to the Isolation Unit of the National Institute for Infectious Diseases ‘Lazzaro Spallanzani’ for isolation and clinical assessment, with a transient mild conjunctivitis and a body temperature of 37.3 °C. Laboratory findings were all within normal values and common respiratory pathogens (influenzavirus A and B, parainfluenza virus, respiratory syncytial virus, rhinovirus, adenovirus, enterovirus, metapneumovirus, bocavirus, coronavirus strains HKU1, NL63, 229E and OC43, *Bordetella pertussis, Mycoplasma pneumoniae* and *Legionella pneumophila*) on pharyngeal swab were excluded. At admission, a second positive real-time RT-PCR on a nasopharyngeal swab confirmed the COVID-19 diagnosis. As the patient was paucisymptomatic, we retrospectively tested serum samples collected at admission using in house-prepared immunofluorescence (IF) slides and neutralisation test as confirmatory test. The IF results showed positivity for both IgG and IgM (≥ 1:640 and 1:80, respectively) at the same time point of the first viral RNA positive result. A preliminary evaluation of the IF test was performed using residual negative samples and few serum samples positive for other human coronaviruses. Two chest computed tomography (CT) scans of the patient (on 7 and 17 February) were normal.

On 7 February, off-label oral treatment with lopinavir/ritonavir (400/100 mg every 12 h) was started after obtaining written informed consent [7]. SARS-CoV-2 RNA in stools, nasopharyngeal and oropharyngeal swabs resulted positive at different time points, whereas urine, spermatic fluid, saliva, blood and conjunctival swabs were persistently negative (Table).

**Table:** Virological and clinical characteristics, COVID-19 patient, Italy, February 2020

February	**5**	**6**	**7**	**8**	**9**	**10**	**11**	**12**	**13**	**14**	**15**	**16**	**17**	**18**
Real-time RT-PCR SARS-CoV-2 RNA														
Naso- and oropharyngeal swab	+	+	+	+	+	+	+	−	−	+	+	+	−	−
Stool Sample	NA	NA	+	+	NA	+	+	+	+	−	−	−	−	−
Clinical characteristics														
BT (°C)	36.5	37.3	37.1	37.1	36.8	37.1	37	36.8	36.6	36.7	36.8	36.9	36.2	36.1
Signs/ symptoms	−	Mild conjunctivitis	−	−	−	−	−	−	−	Tonsillar exudate	Tonsillar exudate	−	−	−
LPV/r treatment	−	−	+	+	+	+	+	+	+	+	+	+	+	+

+: positive; −: negative; BT: body temperature; COVID-19: coronavirus disease 2019; LPV/r: lopinavir/ritonavir; NA: not available; SARS-COV-2: SARS-coronavirus-2.

On 12 and 13 February, two nasopharyngeal swabs resulted negative for SARS-CoV-2 RNA. Nevertheless, on 14 February, a transient whitish exudate on the right palatal tonsil was observed and the oropharyngeal swabs were negative for bacterial growth and again positive for SARS-CoV-2 RNA. No other laboratory finding was remarkable. The exudate disappeared within 48 h.

On 22 February, the patient continued to be asymptomatic and afebrile; the isolation regimen was stopped and the patient discharged.

No other COVID–19 case was observed in the 55 Italian citizens at quarantine. They were discharged at the end of 14-day quarantine after obtaining two SARS-CoV-2 negative samples 24 hours apart near or on the completion of the period.

## Discussion

We present a detailed report of a paucisymptomatic COVID-19 patient with biological samples consecutively collected from different sites. To our knowledge, a previous asymptomatic case in a 10-year old boy, part of a COVID-19 family cluster, with ground-glass lung opacities at chest CT scan has been reported [8].

Our patient was tested for SARS-CoV-2 within the WHO contact protocol applied to the 56 Italians medically evacuated from Wuhan. Otherwise, with no clear exposure or direct contact with possible sources of SARS-CoV-2 infection, he would not have been included in the COVID-19 case definition and not considered for viral screening [5]. Moreover, considering the limited data available at the time on treatment and clinical outcome in COVID-19 patients, an early and off-label treatment with oral lopinavir/ritonavir was started and it could have affected the natural course of the disease. 

A Chinese familial cluster of five patients with COVID-19 pneumonia infected by an asymptomatic carrier has also been described [9]. Two further cases have been reported who transmitted virus during the pre-symptomatic phase and eventually developed fever and respiratory symptoms [10]. It is important to highlight that in our case, the patient, still undiagnosed, did probably not transmit the SARS-CoV-2 infection to his asymptomatic Chinese close contacts and to the other 55 Italian citizens.

Finally, the patient presented persistent viral shedding in stools despite the lack of any gastrointestinal signs and symptoms. In a previous study, the presence of SARS-CoV-2 RNA in faeces was associated with gastrointestinal symptoms [11]. The evidence of extrapulmonary detection of viral RNA in both symptomatic and asymptomatic subjects is of clinical interest in terms of pathogenesis and routes of transmission. However, our results are limited to the molecular detection that does not necessarily mean that infectious virus was present. Additional studies, such as virus culture, should be done to investigate potential infectiousness.

According to the recommendations from WHO, the United States Centers for Disease Control and Prevention and the European Center for Disease Prevention and Control, at least two sequential respiratory tract specimens, collected at least 24 h apart, are required for discharge criteria in clinically recovered patients [12-14]. Nevertheless, our findings suggest that, even in paucisymptomatic patients, consecutive multiple-site biological samples may be required in settings with limited community transmission for discontinuation of infection control and transmission-based isolation precautions.

## Conclusion

This paucisymptomatic case with detailed clinical and virological results is likely to impact on the clinical management of COVID-19 in settings with limited community transmission. Further studies are needed to better understand the clinical spectrum of COVID-19 at hospital and community levels, the role of pauci-/asymptomatic subjects in viral transmission and the clinical relevance of viral persistence in non-respiratory samples as potential sources of SARS-CoV-2 spread.

## References

[1] Tan W Zhao X Ma X Wang W Niu P Xu W A novel coronavirus genome identified in a cluster of pneumonia cases — Wuhan, China 2019−2020. China CDC Weekly. 2020;2:61-2. PMC8393069 34594763

[2] European Centre for Disease Prevention and Control (ECDC). COVID-19. Situation update worldwide. Stockholm: ECDC. [Accessed 17 Mar 2020]. Available from: https://www.ecdc.europa.eu/en/geographical-distribution-2019-ncov-cases

[3] Chen N Zhou M Dong X Qu J Gong F Han Y Epidemiological and clinical characteristics of 99 cases of 2019 novel coronavirus pneumonia in Wuhan, China: a descriptive study. Lancet. 2020;395(10223):507-13. 10.1016/S0140-6736(20)30211-7 32007143 PMC7135076

[4] Wang D Hu B Hu C Zhu F Liu X Zhang J Clinical characteristics of 138 hospitalized patients with 2019 novel Coronavirus-infected pneumonia in Wuhan, China. JAMA. 2020. [Epub ahead of print]. 10.1001/jama.2020.1585 32031570 PMC7042881

[5] World Health Organization (WHO). The First Few X (FFX) Cases and contact investigation protocol for 2019-novel coronavirus (2019-nCoV) infection. Geneva: WHO; 29 Jan 2020. Available from: https://www.who.int/publications-detail/the-first-few-x-(ffx)-cases-and-contact-investigation-protocol-for-2019-novel-coronavirus-(2019-ncov)-infection

[6] Corman VM Landt O Kaiser M Molenkamp R Meijer A Chu DKW Detection of 2019 novel coronavirus (2019-nCoV) by real-time RT-PCR. Euro Surveill. 2020;25(3):2000045. 10.2807/1560-7917.ES.2020.25.3.2000045 31992387 PMC6988269

[7] Li G De Clercq E Therapeutic options for the 2019 novel coronavirus (2019-nCoV). Nat Rev Drug Discov. 2020;19(3):149-50. 32127666 10.1038/d41573-020-00016-0

[8] Chan JFW Yuan S Kok KH To KKW Chu H Yang J A familial cluster of pneumonia associated with the 2019 novel coronavirus indicating person-to-person transmission: a study of a family cluster. Lancet. 2020;395(10223):514-23. 31986261 10.1016/S0140-6736(20)30154-9 PMC7159286

[9] Bai Y Yao L Wei T Tian F Jin DY Chen L Presumed asymptomatic carrier transmission of COVID-19. JAMA. 2020. [Epub ahead of print]. 10.1001/jama.2020.2565 32083643 PMC7042844

[10] Rothe C Schunk M Sothmann P Bretzel G Froeschl G Wallrauch C Transmission of 2019-nCoV infection from an asymptomatic contact in Germany. N Engl J Med. 2020;382(10):970-1. 10.1056/NEJMc2001468 32003551 PMC7120970

[11] Holshue ML DeBolt C Lindquist S Lofy KH Wiesman J Bruce H First case of 2019 novel coronavirus in the United States. N Engl J Med. 2020;382(10):929-36. 10.1056/NEJMoa2001191 32004427 PMC7092802

[12] World Health Organization (WHO). Clinical management of severe acute respiratory infection when novel coronavirus (2019-nCoV) infection is suspected. Interim guidance. Geneva: WHO; 28 Jan 2020. Available from: https://www.who.int/publications-detail/clinical-management-of-severe-acute-respiratory-infection-when-novel-coronavirus-(ncov)-infection-is-suspected

[13] Centers for Disease Control and Prevention (CDC). Interim guidance for discontinuation of transmission-based precautions and disposition of hospitalized patients with COVID-19. Atlanta: CDC. [Accessed: 16 Mar 2020]. Available from: https://www.cdc.gov/coronavirus/2019-ncov/hcp/disposition-hospitalized-patients.html

[14] European Centre for Disease Prevention and Control (ECDC). Novel coronavirus (SARS-CoV-2). Technical Report. Discharge criteria for confirmed COVID-19 cases – When is it safe to discharge COVID-19 cases from the hospital or end home isolation? Stockholm: ECDC. [Accessed: 16 Mar 2020]. Available from: https://www.ecdc.europa.eu/sites/default/files/documents/COVID-19-Discharge-criteria.pdf

